# Targeted Drug Administration onto Cancer Cells Using Hyaluronic Acid–Quercetin-Conjugated Silver Nanoparticles

**DOI:** 10.3390/molecules28104146

**Published:** 2023-05-17

**Authors:** Rasha H. Al-Serwi, Mohamed A. Eladl, Mohamed El-Sherbiny, Mohamed A. Saleh, Gamal Othman, Sultan M. Alshahrani, Rasha Alnefaie, Afnan M. Jan, Sulaiman M. Alnasser, Aishah E. Albalawi, Jamal Moideen Muthu Mohamed, Farid Menaa

**Affiliations:** 1 Department of Basic Dental Sciences, College of Dentistry, Princess Nourah bint Abdulrahman University, Riyadh 11671, Saudi Arabia; 2 Department of Basic Medical Sciences, College of Medicine, University of Sharjah, Sharjah 27272, United Arab Emirates; 3 Department of Basic Medical Sciences, College of Medicine, AlMaarefa University, Riyadh 11597, Saudi Arabia; 4 Department of Anatomy and Embryology, Faculty of Medicine, Mansoura 35511, Egypt; 5 Department of Clinical Sciences, College of Medicine, University of Sharjah, Sharjah 27272, United Arab Emirates; 6 Department of Pharmacology and Toxicology, Faculty of Pharmacy, Mansoura University, Mansoura 35516, Egypt; 7 Department of Clinical Pharmacy, College of Pharmacy, King Khalid University, Abha 61441, Saudi Arabia; 8 Department of Biology, Faculty of Science, Al-Baha University, Al Baha 65779, Saudi Arabia; 9 Department of Biochemistry, Faculty of Medicine, Umm Al-Qura University, Makkah 21955, Saudi Arabia; 10 Department of Pharmacology and Toxicology, Unaizah Colleage of Pharmacy, Qassim University, Buraydah 52571, Saudi Arabia; 11 Department of Biology, Faculty of Science, University of Tabuk, Tabuk 47913, Saudi Arabia; 12 Vaasudhara College of Pharmacy, Rajiv Gandhi University of Health Sciences, Sante Circle, Chintamani Road, Hoskote 562114, Karnataka, India; 13 Departments of Medicine and Nanomedicine, California Innovations Corporation, San Diego, CA 92037, USA

**Keywords:** water-soluble quercetin, silver nanoparticles, functional polymer–drug conjugates, nanodrug delivery systems, hemocompatibility, cytotoxicity, oncotargets

## Abstract

Quercetin (QtN) displays low systemic bioavailability caused by poor water solubility and instability. Consequently, it exerts limited anticancer action in vivo. One solution to increase the anticancer efficacy of QtN is the use of appropriate functionalized nanocarriers that preferentially target and deliver the drug to the tumor location. Herein, a direct advanced method was designed to develop water-soluble hyaluronic acid (HA)-QtN-conjugated silver nanoparticles (AgNPs). HA-QtN reduced silver nitrate (AgNO_3_) while acting as a stabilizing agent to produce AgNPs. Further, HA-QtN#AgNPs served as an anchor for folate/folic acid (FA) conjugated with polyethylene glycol (PEG). The resulting PEG-FA-HA-QtN#AgNPs (further abbreviated as PF/HA-QtN#AgNPs) were characterized both in vitro and ex vivo. Physical characterizations included UV-visible (UV-Vis) spectroscopy, Fourier transform infrared (FTIR) spectroscopy, transmission electron microscopy (TEM), particle size (PS) and zeta potential (ZP) measurements, and biopharmaceutical evaluations. The biopharmaceutical evaluations included analyses of the cytotoxic effects on the HeLa and Caco-2 cancer cell lines using the MTT assay; cellular drug intake into cancer cells using flow cytometry and confocal microscopy; and blood compatibility using an automatic hematology analyzer, a diode array spectrophotometer, and an enzyme-linked immunosorbent assay (ELISA). The prepared hybrid delivery nanosystem was hemocompatible and more oncocytotoxic than the free, pure QtN. Therefore, PF/HA-QtN#AgNPs represent a smart nano-based drug delivery system (NDDS) and could be a promising oncotherapeutic option if the data are validated in vivo.

## 1. Introduction

Targeting cancer cells with precision represents one of the major challenges in chemotherapy. Unfortunately, conventional cancer chemotherapy often damages healthy tissues while acting relatively well on cancer cells. In addition, the hydrophobic nature of many anticancer drugs limits the use of conventional delivery methods [1].

Multidrug resistance (MDR), which arises from conventional cytotoxic (chemical and/or biological) drugs, remains another obstacle to overcome in cancer chemotherapy. The drawbacks of conventional drug delivery systems include a lack of targeting, nonspecific biodistribution, a lack of aqueous solubility, low therapeutic indices, and poor oral bioavailability. In this regard, nanotherapeutics represent a solution to overcome these drawbacks. Effective NDDSs are usually made from diverse materials with different chemical compositions that are tailored to carry one or more therapeutic agents (e.g., anticancer drugs) for controlled and targeted delivery [2].

AgNPs have been widely investigated because of their unique properties, such as biocompatibility, customizable surface characteristics for functionalization, easy synthesis, and stability under most in vivo conditions (e.g., a longer half-life in the blood circulation) [3]. The surface characteristics of AgNPs may be completely controlled for the targeted and sustained release of a given bioactive compound (e.g., chemicals and biologicals) [4].

Quercetin (QtN) is widely present in a variety of fruits and vegetables, including apples, lovage, capers, dill, onions, cilantro, and different berries (e.g., cranberries, lingonberries, and chokeberries). It is generally known that QtN exerts anticarcinogenic activities by blocking enzymes and/or attaching to proteins and cellular receptors involved in carcinogenesis [5]. QtN is a secondary metabolite of flavonol and belongs to the family of flavonoids. It contains a benzo-(γ)-pyrone skeletal structure, represented by C6-C3-C6 (carbon structure), made up of double benzene rings, A and B, connected by a three-carbon pyrone ring, C. It has five hydroxyl groups at carbons 3, 3′, 4′, 5, and 7. QtN is therefore also known as a pentahydroxy flavonol [6]. The comparative replacement of different functional groups on the flavonol molecule enables QtN and its metabolites to act in a wide range of biochemical and pharmacological activities. QtN specifically inhibited the growth of cancer cells by arresting the cells in the G1 phase (gap 1). Indeed, a low amount of QtN-induced Chk2 (checkpoint kinase 2) activation increased the activity of the p21 CDK (cyclin-dependent kinase) inhibitor, which trapped E2F1 (E2F transcription factor 1) and slowed the advancement of the G1/S cell cycle, leading to modest DNA damage. Moreover, cyclin B_1_ (encoded by the CCNB1 gene in humans) and CDK, two crucial regulators of the G2/M cell cycle, were found to be downregulated by QtN, which prevented the transcription factor NF-Y from binding to the promoter of the cyclin B1 gene and led to transcription inhibition [7].

The inherent lipophilicity and instability of QtN in blood circulation have a significant negative impact on in vivo bioavailability, substantially restricting its practical usage.

Researchers have suggested various strategies to circumvent the unspecific action and poor water-solubility of QtN. Among them, QtN conjugated to water-soluble polymer chain ends or side chains has proven to be successful. The most widely used polymers include chitosan (CS), polyvinylpyrrolidone (PVP), and polyethylene glycol (PEG). Studies demonstrated that the cytotoxicity of the polymer-based QtN conjugates increased compared to the free, pure QtN [8].

HA (also called hyaluronan), a naturally occurring polysaccharide, is specifically an anionic non-sulfated glycosaminoglycan that is distributed widely throughout the connective, epithelial, and neural tissues. HA is a polymer consisting of repeating units of D-N-acetylglucosamine and D-glucuronic acid linked together via alternating beta-1,4 and beta-1,3 glycosidic bonds. Importantly, HA gained much interest in oncology (angiogenesis) and pharmacology (the design of innovative therapeutics) because its cell-specific surface markers, including the receptor for hyaluronan-mediated motility (RHAMM) and cluster of differentiation 44 (CD44), are heavily expressed in many different types of cancers [9,10]. As a result, malignant cells with strong metastatic activity frequently have elevated HA binding and uptake [9]. Mechanistically, the polysaccharide backbone of HA contains a moiety that can specifically recognize CD44. CD44 is a transmembrane receptor that is overexpressed on the surfaces of various cells, including those of pancreatic cancer, and is also involved in metastatic spread and cancer aggressiveness [11]. Recently, a tumor-cell-targeted prodrug was developed for QtN, using HA as a polymeric carrier. HA-QtN bioconjugates were synthesized by linking the hydroxy of QtN via a succinate ester to adipic dihydrazide-modified HA [12]. The resulting HA-QtN micelles exhibited significant sustained and pH-dependent drug release behaviors without dramatic initial bursts. Compared to a free QtN solution, the HA-QtN micelles were four times more cytotoxic to MCF-7 cells (a CD44-overexpressing cell line) compared to L929 cells (a CD44-deficient cell line) [12]. The HA-QtN micelles also showed an excellent inhibition effect on tumor growth in H22 tumor-bearing mice [12]. An analysis of hemolytic toxicity and a vein irritation assay further suggested that the HA-QT micelles were a safe and potent drug delivery system for targeted antitumor therapy [12]. The roles of HA in improving the solubility and permeability of active pharmaceutical ingredients, enhancing emulsion properties, prolonging stability, reducing immunogenicity, and providing targeting have been well reviewed recently [13].

Numerous studies have shown that coating NPs with PEG can lessen the opsonization on their surface and lengthen the time that they are circulated in the blood, and FA was selected as one of the targeted molecules [14]. A high-affinity glycosyl phosphatidylinositol (GPI)-anchored protein called the folate receptor is overexpressed in diverse human tumor types, such as ovarian, kidney, uterine, and colon adenocarcinomas. Highly selective locations that distinguish tumor cells from normal cells are provided by folate receptors. FA acts as a ligand for nanocarriers to target cancer cells and facilitates their entrance via folate-receptor-mediated endocytosis [15].

The pertinence of the present study resides both in enhancing the oncocytotoxic action of QtN and maximizing/optimizing its bioavailability profile. Therefore, we hypothesized that PF/HA-QtN#AgNPs could be an original and effective NDDS to target cancers. QtN was loaded into multifunctional biodegradable AgNPs conjugated to FA-PEG (PF), which were externally decorated with HA. Therefore, QtN could stabilize AgNPs, while HA would allow better tumor cell targeting, and PF would circumvent the poor water-solubility and increase the cytotoxicity.

Although there have been numerous studies on the use of functionalized AgNPs for drug delivery, there has been a paucity of investigations on the production and stabilization of AgNPs utilizing polymer–anticancer drug conjugates. Additionally, we are not aware of any reports describing the covalent immobilization of a water-soluble QtN conjugate on AgNPs. The novelty of this study is then further based on an innovative approach that involves the covalent attachment of QtN to AgNPs, followed by the functionalization of QtN-AgNPs with PEG-FA. Such alterations would enable particles with improved tumor targeting thanks to the joint effects of FA and HA on endocytosis and enhanced permeability and retention (EPR).

In the present study, PF/HA-QtN#AgNPs were mainly evaluated for their physicochemical qualities, blood compatibility, and effectiveness in targeting and eliminating cancer cells.

## 2. Results

### 2.1. Synthesis and Physicochemical Properties of AgNPs

**Figure 1:**
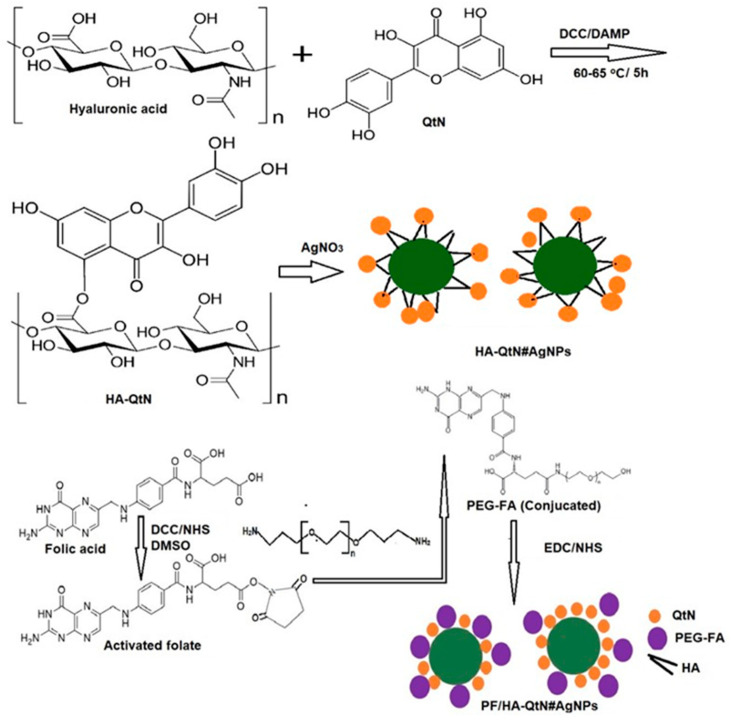
Schematic representation of PF/HA-QtN#AgNP generation. QtN: Quercetin; HA: Hyaluronic acid; AgNPs: Silver nanopartilces; AgNO_3_: Silver nitrate; FA: Folic acid; PEG; Polyethylene glycol; DMSO: Dimethylsulfoxide; DCC: 1, 3-dicyclohexylcarbodiimide; DAMP: 4-dimethylaminopyridine; EDC: 1-ethyl-3-(3-dimethylaminopropyl) carbodiimide, NHS: *N*-hydroxysuccinimide.

Figure 1 shows the synthesis pathway of PF/HA-QtN#AgNPs. Following this phase, the plasmon absorbance did not expand noticeably, showing that PF/HA-QtN#AgNPs did not aggregate in the colloidal suspension. AgNPs with controlled PS and shape require stabilizing agents, reductants, and drug molecules. To conjugate the drug onto AgNPs, HA-QtN was used to prepare HA-QtN#AgNPs in a single step. Biogenic AgNPs are typically dark brown (after a color change from light brown), and the intensity and size of the NPs affect the color [16].

**Figure 2:**
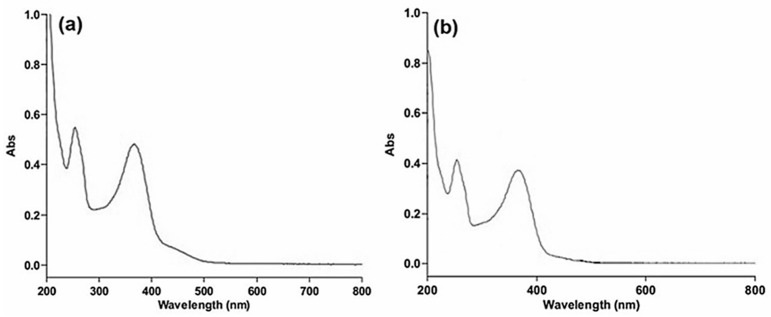
UV–Vis spectra of (**a**) HA-QtN#AgNPs and (**b**) PF/HA-QtN#AgNPs.

UV-Vis spectroscopy is an important method for verifying the synthesis and durability of AgNPs in water-based solutions. Within 30 min of gradual heating, a change in color from a transparent/colorless solution to yellowish brown was observed upon the addition of an aqueous HA-QtN solution into an aqueous AgNO_3_ solution; this shift in color indicated the synthesis of HA-QtN#AgNPs. Figure 2a shows the typical absorption peak of HA-QtN#AgNPs. The change in color was due to surface plasmon resonance (SPR) excitation, which is commonly examined using a UV-Vis spectrophotometer. The SPR absorption band was due to the collective vibration of free e^−^ of metal NPs in resonance with light waves [4].

To increase the targeting ability of HA-QtN#AgNPs, FA was conjugated on their surfaces. By coupling carboxyl groups with the amino group of PEG-FA utilizing EDC chemistry, FA was conjugated on HA-QtN#AgNPs. Every stage of the modification involved monitoring variations in the plasmon resonance absorption. The spectra show that the SPR peak of the AgNPs was detected at 354 nm, with a significant level of absorption (Figure 2a), which is a distinctive characteristic of AgNPs [17]. PF/HA-QtN#AgNPs displayed an absorption peak at about 367 nm (Figure 2b). The chemisorption of PEG-FA caused a change in the dielectric constant around the NPs, which resulted in a red shift in the SPR peak. The UV-Vis spectrum profile of HA-QtN#AgNPs was quite similar to that of PF/HA-QtN#AgNPs. The analysis of the spectra of both HA-QtN#AgNPs indicated that the absorption did not increase further, which indicates that the reaction (PEG-FA) was completed. The stability of the reaction mixture was also examined, and the AgNPs maintained the same peak wavelength and absorption intensity throughout the experiment.

Table 1 displays the PS values obtained from DLS measurements of HA-QtN#AgNPs and PF/HA-QtN#AgNPs in DI water devoid of any filtration at 25 °C. According to the DLS data, the hydrodynamic diameter of the HA-QtN#AgNPs was, on average, 72.11± 1.31 nm. The PF/HA-QtN#AgNPs had an average hydrodynamic diameter of 131.22 ± 3.51 nm. The magnitude of the particles’ ZP is also shown in Table 1. For HA-QtN#AgNPs, the ZP was -42.33 ± 1.67 mV. This ZP produced a sufficient surface charge to prevent particle aggregation. When PEG-FA (PF) was conjugated to the surfaces of HA-QtN#AgNPs, the ZP was lowered to -28.9 ± 1.98 mV. PF/HA-QtN#AgNPs possess an adequate repulsive force to avoid aggregation during storage (long-term), even though the surface charge is somewhat diminished.

**Figure 3:**
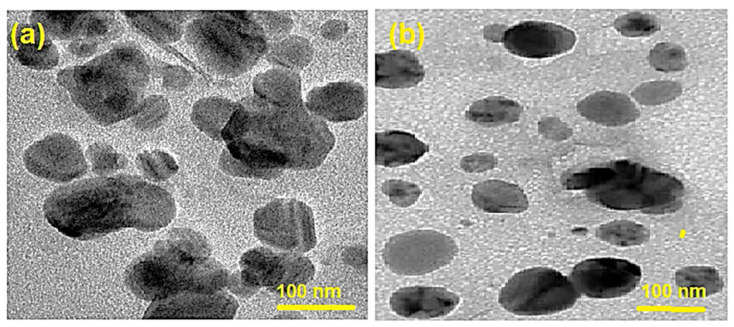
TEM images of (**a**) HA–QtN#AgNPs and (**b**) PF/HA–QtN#AgNPs. The scale bars indicate 100 nm.

These findings are in line with the TEM observations (Figure 3). TEM images of HA-QtN#AgNPs and PF/HA-QtN#AgNPs are depicted in Figure 3a,b, respectively. TEM scans revealed particles in a more scattered form without aggregation. The HA-QtN#AgNPs and PF/HA-QtN#AgNPs were found to be 24.0 ± 3.1 nm and 33.6 ± 4.1 nm in diameter, respectively.

### 2.2. Structural (Functional Chemical Group) Analysis Using FTIR

**Figure 4:**
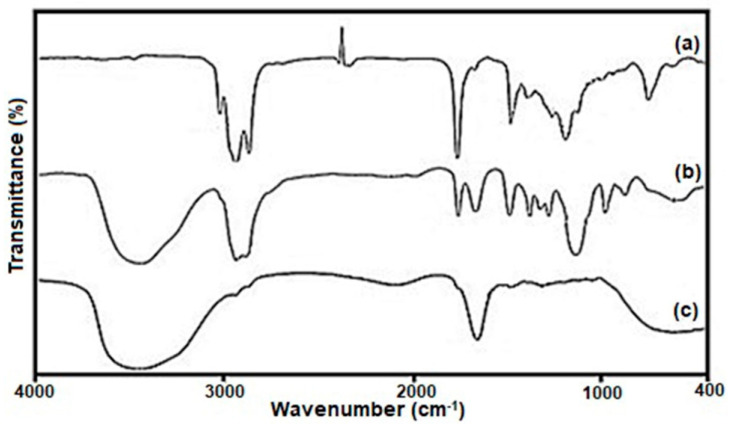
FTIR bands of (**a**) HA−QtN#AgNPs, (**b**) PEG−FA, and (**c**) PF/HA−QtN#AgNPs.

FTIR was used to analyze the surfaces of HA-QtN#AgNPs enhanced with PEG-FA (Figure 4). The FTIR assignments of HA-QtN#AgNPs, PEG-FA (PF), and PF/HA-QtN#AgNPs are summarized in Table 2.

The OH stretching frequency was given to a large band in the first spectrum at roughly 3008 cm^−1^. Peaks near 1745 cm^−1^ were caused by carboxylate anions (COO^−^) being stretched by C-O. Due to the C-O stretching frequency (ester linkages) in the HA-QtN conjugate, a band could be detected at about 1375 cm^−1^. Two distinct peaks at 2926 cm^−1^ were explained by the HA-QtN molecules being stretched by aliphatic CAH bonds on the surfaces of the AgNPs. Moreover, the one inverted peak related to CO_2_ is shown in Figure 4a. This is common, as the COO^−^ concentration usually fluctuates during measurements in an open chamber.

The chemical conjugation of PEG-FA (Figure 4b) on the surfaces of HA-QtN#AgNPs (Figure 4a) by amide linkage was successfully demonstrated by the appearance of peaks ascribed to the C=O stretching vibration at 1735.65 cm^−1^ and N-H bending vibration at 1462 cm^−1^ in the FTIR spectrum of PF/HA-QtN#AgNPs (Figure 4c). The combined effects of the free carboxylate anion on HA-QtN#AgNPs, the free NH_2_ group on PF/HA-QtN#AgNPs, and the OH stretching vibration of the free carboxylic acid group in PEG-FA were attributed to a bandwidth at approximately 3446.17 cm^−1^ (Table 2). The peak at 1637 cm^−1^ was attributed to the free NH_2_ group of folates on PF/HA-QtN#AgNPs (NH bending vibration). Both HA-QtN#AgNPs and PEG-FA displayed strong bands at around 2900 cm^−1^ for N-H stretching (Figure 4a,b); however, the bands nearly disappeared in this region for PF/HA-QtN#AgNPs because QtN and PEG-FA were completely conjugated within the NP core. More NH_2_ groups appeared to have been converted to N-H groups, according to the PF/HA-QtN#AgNPs (NH in secondary amides).

### 2.3. In Vitro Hemocompatibility Behavior

In vitro hemolysis is a quick and accurate method for determining blood compatibility. The results show that PF/HA-QtN#AgNPs induced hemolysis as low as 0.052 ± 0.0041%. The compatibility of PF/HA-QtN#AgNPs with blood was verified using platelet aggregation and activation assays.

After blood exposure, the data demonstrated a decrease in the aggregation response to the agonists ADP and collagen. Indeed, following exposure to PF/HA-QtN#AgNPs, there were decreases in platelet aggregation in response to both agonists (31.4 ± 1.6 and 9.9 ± 0.3% for ADP and collagen, respectively). Due to the compatible behavior of PF/HA-QtN#AgNPs, the platelets suspended in PF/HA-QtN#AgNPs did not aggregate.

The hemocompatibility of PF/HA-QtN#AgNPs was once more validated utilizing the platelet alpha granule secretion (PF4) assay. PF/HA-QtN#AgNPs were exposed to platelet-deficient plasma (PPP) to test the platelet activation affinity, and an ELISA kit was used to measure the percentage change in platelet secretion factor PF4. The outcome demonstrated that there was no difference in the PF4 concentration prior to and following exposure to the substance.

These findings showed that PF/HA-QtN#AgNPs are hemocompatible, pointing to their potential for intravenous (IV) injection.

### 2.4. In Vitro Cytotoxicity Studies

**Figure 5:**
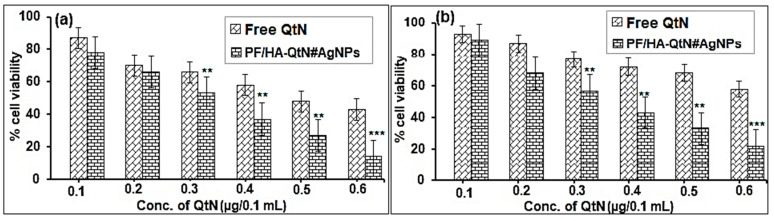
Cell viability of (**a**) HeLa cells and (**b**) Caco-2 cells in PF/HA-QtN#AgNPs and free QtN at 0.1 to 0.6 µg/0.1 mL concentrations (means ± SDs, n = 3). Significant variances associated with the control (free QtN) are denoted by ** (*p* < 0.05) and *** (*p* < 0.005), as estimated by the ANOVA test.

In a further step, we investigated how PF/HA-QtN#AgNPs and free QtN affected HeLa and Caco-2 cells. Cytotoxicity was measured following a 24 h incubation with functionalized AgNPs or free QtN at escalating concentrations, and the data are reported in Figure 5.

After cell incubation in a medium containing 0.22–0.45 µg/0.1 mL of conjugated QtN, it was discovered that the viability of HeLa cells (Figure 5a) and Caco-2 cells (Figure 5b) ranged from 81 to 15 percent and 83 to 26%, respectively. When exposed to equal amounts of free QtN, the two cell lines (range of 0.1–0.6 µg/0.1 mL) produced cell viability readings between 91 and 43% for HeLa cells and between 95 and 62% for Caco-2 cells (Figure 5b). As the concentrations increased, the effects of both conjugated and free QtN on cell survival declined. However, PF/HA-QtN#AgNPs showed that the cytotoxicity was markedly enhanced (*p* < 0.05) compared to that of free QtN (control).

### 2.5. Cellular Uptake

**Figure 6:**
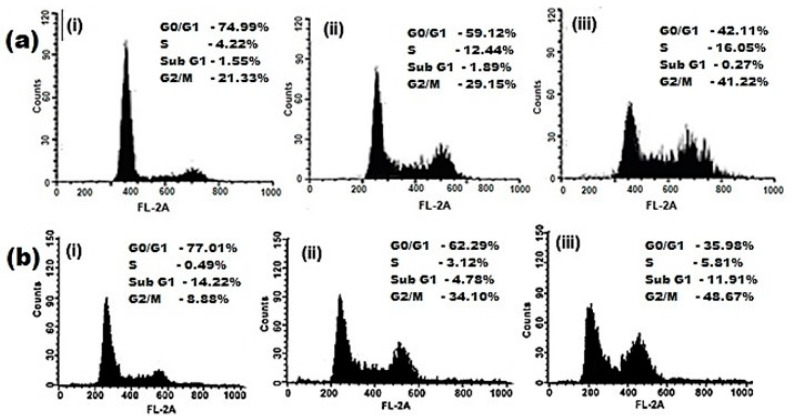
Flow cytometry images of (**a**) Caco-2 cells and (**b**) HeLa cells of (**i**) control (the absence of AgNPs), (**ii**) cells treated with HA-QtN#AgNPs, and (**iii**) cells treated with PF/HA–QtN#AgNPs.

FACS was used to evaluate the effect of the cellular uptake behavior of PF/HA-QtN#AgNPs in folate-receptor-positive Caco-2 (Figure 6a) and HeLa (Figure 6b) cells. Flow cytometry histograms obtained from Caco-2 and HeLa cells incubated with PF/HA-QtN#AgNPs (test) or HA-QtN#AgNPs (control) are shown in Figure 6. To control for autofluorescence, control cells were not incubated with AgNPs (negative control).

In Caco-2 and HeLa cells, the uptake of HA-QtN#AgNPs was 29.15% and 34.10%, respectively, whereas the uptake of PF/HA–QtN#AgNPs was 41.32 and 48.67%, respectively. The increased cellular absorption of PF/HA-QtN#AgNPs compared to HA-QtN#AgNPs may have been caused by the substantial concentration of FA on their surfaces.

**Figure 7:**
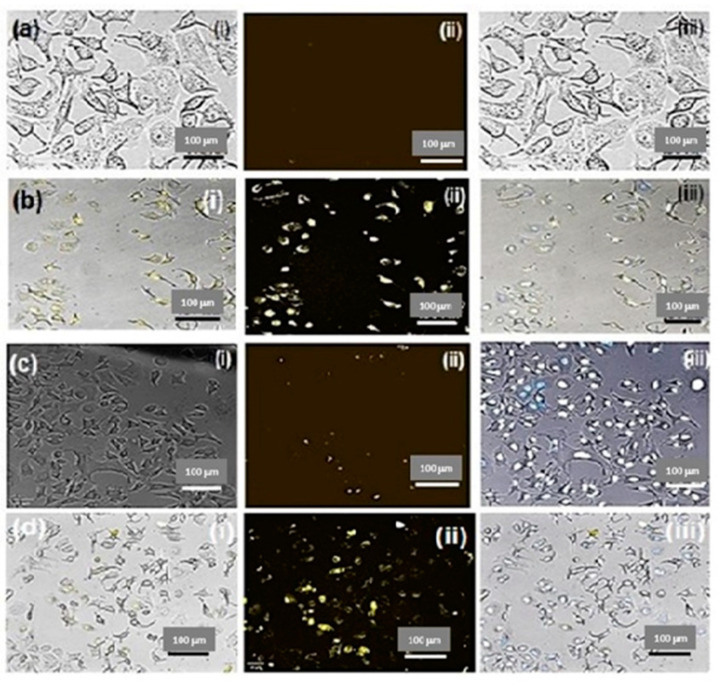
Drug localization studies after 4 h of incubation of (**a**) control (untreated Caco-2 cells), (**b**) Caco-2 cells incubated with PF/HA–QtN#AgNPs, (**c**) control (untreated HeLa cells), and (**d**) HeLa cells incubated with PF/HA–QtN#AgNPs. The images are shown as (**i**) bright field, (**ii**) fluorescence, and (**iii**) merged images. The scale bars indicate 100 μm. The magnification is 10×.

To further describe the cellular uptake behavior and analyze the intracellular distribution, CLSM was used (Figure 7). The Caco-2 cells incubated with PF/HA-QtN#AgNPs for 4 h displayed brighter intracellular fluorescence (Figure 7b) compared to HeLa cells (Figure 7d). As expected, the corresponding control cells (untreated) did not display detectable fluorescence (Figure 7a,c). These data are in accordance with the cellular uptake behavior seen by FACS. The intake of PF/HA-QtN#AgNPs into Caco-2 cells was higher compared to that of HeLa cells, which strongly suggests that Caco-2 cells represent a better model for such an assay. The internalization behavior of PF/HA-QtN#AgNPs was examined using fluorescence microscopic images that were obtained after nuclear labeling. The images unambiguously demonstrated that modified NPs were present in the perinuclear regions as well as in the nuclei. 

Taken together, the antiproliferative impact and nuclear localization make PF/HA-QtN#AgNPs a promising candidate for cancer chemotherapy.

## 3. Discussion

QtN is an anticancer phytochemical that is hydrophobic and extremely unstable in aqueous media [7]. It has been demonstrated that conjugating QtN with the naturally occurring polysaccharide HA can considerably increase QtN’s stability, solubility, and oncotargeting [10]. Moreover, PEG-FA can be used to target cancer cells effectively [18].

Therefore, in this pioneering study, we successfully synthesized AgNPs and produce unaggregated, stable, and water-soluble QtN-conjugated AgNPs by employing HA-QtN and PEG-FA. QtN molecules were conjugated with NPs in a significant way using the surface chemistry of AgNPs. QtN was used as a natural/green and non-toxic reducing and capping agent in the present study. An additional stabilizing agent was not required for HA-QtN#AgNPs because they were found to be stable after being stored at 20 °C for more than three months. UV-Vis and FTIR confirmed the successful synthesis of the NDDS. The interactions of the main active groups of the compounds involved in the NDDS were evidenced by FTIR. TEM and DLS confirmed a PS in the nanoscale range and showed well-dispersed NPs. It is worth noting that the disparity in PS values between measurements made using DLS and TEM is often seen when a hydrophilic part is deposited on an NP’s surface [19]. During the DLS tests, the hydrophilic coating increased the average hydrodynamic diameter, in accordance with previous studies [19].

To the best of our knowledge, this is the first report about the synthesis and characterization of this NDDS, namely PF/HA-QtN#AgNPs. Several works have described in detail the synthesis and characterization of HA-QtN, and it was discovered that there were 1.5 ± 0.4 mg of QtN in every 100 mg of the HA-QtN conjugate [10,12]. The major factors to consider for an effective treatment using such an NDDS include aggregation prevention; the oncocytotoxic potential; the increased circulation duration (systemic half-life); the capacity to target the tumor site in a controlled manner; the obtention of the necessary load; and the compatibility with the body fluids (e.g., blood) and healthy cells, organs, and immune system [17,20,21,22].

Importantly, PF/HA-QtN#AgNPs were found to be hemocompatible based on a hemolytic assay and platelet activation affinity [23,24]. Furthermore, since most of the cancer cells overexpressed folate receptors, which allow the active adsorption of these NPs through endocytosis and the EPR effect [15], the higher onco-cytotoxicity observed with PF/HA-QtN#AgNPs compared to free QtN was likely caused by higher uptake due to improved cellular internalization through the folate-receptor-mediated endocytosis of the NDDS [22]. Indeed, it was shown that the target uptake efficiency varies depending on the amount of folate receptors present in different colorectal cancer cell lines [25]. The flow cytometry and CLSM investigations supported this statement. The microscopic examinations unequivocally showed that functionalized particles were found in the perinuclear space as well as the nucleus. FA has been considered a promising ligand for improving the cellular endocytosis of medicines and macromolecules into cancer cells [26]. We conjugated FA on the surfaces of HA-QtN#AgNPs because of its unique properties. By joining the amino group of PEG (P) with the carboxyl group of the FA (F) ring using EDC chemistry (coupling mechanism resulting in amide group formation), PF was conjugated on HA-QtN#AgNPs. PF/HA-QtN#AgNPs became even more stable because of the increased hydrophilicity brought on by the addition of PEG (PEGylation), which repelled proteins (opsonins) [22,27].

The data show that PF/HA-QtN#AgNPs are a better onco-chemotherapeutic option compared to free QtN and HA-QtN#AgNPs, based on their capacity to bind, internalize into cancer cells (nuclear localization), and exert their cytotoxic effect/antiproliferative action. The various functional groups on the polymer–drug conjugate allow for the incorporation of other drugs, making the technique incredibly adaptable and allowing for the customization of the system as a multidrug and multifunctional (nano)carrier.

## 4. Materials and Methods

### 4.1. Materials

Poly (ethyleneglycol) bis (3-aminopropyl ether) (PEG 1500); folic acid (FA); silver nitrate; poloxamer 407 (P-407); 1, 3-dicyclohexylcarbodiimide (DCC); 4-dimethylaminopyridine (DMAP); Dimethylsulfoxide (DMSO), 1-ethyl-3-(3-dimethylaminopropyl)carbodiimide/*N*-hydroxysuccinimide (EDC/NHS), Dulbecco’s Minimal essential medium (DMEM), Fetal bovine serum (FBS), and MTT (3-(4, 5-Dimethylthiazol-2yl)-2, 5, diphenyltetrazoliumbromide) reagent were all purchased from Sigma-Aldrich, Bangalore, India. The National Centre for Cell Sciences in Pune, India, provided cancer cell lines such as HeLa (immortalized human cervical carcinoma) and Caco-2 (immortalized human epithelial colorectal adenocarcinoma) cells. Rhodamine B isothiocyanate, Toluidine Blue-O (TBO), Kaighn’s version of Ham’s F12 medium (F12K), Folate-free RPMI 1640 (FFRPMI), Fluorescence isothiocyanate (FITC), and EDTA/Trypsin were bought from BASF Corporation, Mumbai, India.

### 4.2. Synthesis of HA-QtN Conjugates

HA-QtN conjugates were prepared using a previously mentioned method [18]. Briefly, 800 mg of a 1% wt solution of HA, DCC (100 mg), and DMAP was added to a mixture (1:1 *v/v*) of water and DMSO (40 mg). After 1 h of stirring to actuate the carboxylic group of HA, 0.105 mM QtN was dissolved in 25 mL of DMSO and added slowly to the above-mentioned solution while being concealed in a nitrogen chamber. For roughly 6 h, the mixture was vigorously agitated at 60–65 °C. The resulting solution was dialyzed using a membrane (molecular weight cut-off of 3500 KDa) in deionized (DI) water (DIW) versus (vs.) DMSO for 1 day; DIW was used again for 3 days to completely eliminate unbound molecules (Figure 1). The dialysis was followed by lyophilization (Christ, Alpha 1–2 LD plus, Gernsheim, Germany), and the lyophilized HA-QtN conjugates were stored in a cold environment.

### 4.3. Synthesis of AgNPs Using HA-QtN Conjugates

Briefly, HA-QtN in an aqueous solution (40 mg/100 mL) was prepared. First, 50 mL of the solution was added dropwise to 1000 µL of 10^−2^ M AgNO_3_ in an aqueous solution. As the solution was gradually heated, the growth of the AgNPs was monitored, as indicated by the color change of a colorless solution that turned to yellowish brown. To prepare HA-QtN/AgNPs, the reaction mixture was then cooled to room temperature (RT), dialyzed (molecular weight cut-off of 3500 KDa) against DI water for 24 h, and freeze-dried (Figure 1).

### 4.4. PEG-FA Chemisorptions on the Surfaces of HA-QtN/AgNPs

PEG-FA (PF)-conjugated HA-QtN/AgNPs were prepared in two phases. During the first phase, anhydrous DMSO was used to dissolve FA (75 mg, 0.1745 mM), NHS (3.19 mg, 0.1745 mM), and DCC (5.63 mg, 0.1745 mM). At RT, the activation reaction continued for one hr in nitrogen. Filtration was used to remove the insoluble dicyclohexyl urea. PEG (262.5 mg, 0.1745 mM), a heterofunctional PEG derivative, was added to the activated folate mixture [18]. The reaction solution was dialyzed (molecular weight cut-off of 1000 KDa) vs. a DI aqueous solution to eliminate unreacted substrates after 4 h of reaction and was then lyophilized to produce folate-conjugated PEG (PF). In a 5 mM sodium bicarbonate solution (pH: 8.4), the terminal carboxyl groups of HA-QtN/AgNPs (10 mg) were triggered with EDC/NHS for one hr (Figure 1). Subsequently, 0.01 mM PF was added, and stirring was kept constant for approximately 18 h at RT. After dialysis (molecular weight cut-off of 3500 KDa) against DI water and freeze drying the reaction mixture, the modified particles, known as HA-QtN/AgNPs-PEG-FA (abbreviated as PF/HA-QtN#AgNPs), were obtained.

### 4.5. Physical Characterizations

#### 4.5.1. UV-Vis Spectroscopy Analysis

The easiest method to confirm the formation of NPs remains UV-Vis spectroscopy. A Cary 60 UV-Vis spectrometer (Agilent, Santa Clara, CA, USA) was employed. Distilled water (dH_2_O) was used as a reference, and the absorbance spectrum of the AgNP sample was measured in the 200–800 nm region [17].

#### 4.5.2. FTIR Analysis

To assess potential interactions (structural variations) between the medication, the lipid core, and the coating materials, an FTIR analysis was conducted [12,23]. The infrared spectra were obtained using an FTIR-6300 spectrometer (JASCO Corp., Tokyo, Japan). The IR spectra of the solid materials were captured in the solid state using the KBr disc method spanning the wavenumber range of 4000–400cm^−1^ with a scan speed of 1 cms^−1^.

#### 4.5.3. TEM Analysis

TEM was used to observe the morphology of the freshly prepared AgNPs [12,23,28]. Briefly, one drop of the stained sample was placed on a 200-mesh copper grid for 10 min after the samples were first mixed with an equivalent volume of 2% phosphotungstate (pH adjusted to 7). The sample was imaged at 80 kV using an H-7650 TEM (HITACHI, Tokyo, Japan).

#### 4.5.4. PS and ZP Measurements

A Malvern Zetasizer Nano ZS (Malvern Instruments Ltd., Malvern, UK) and a helium–neon (He-Ne) laser beam with a wavelength of 523.1 nm were used to measure the PS and ZP [23,28]. Prior to these analyses, the NPs were diluted appropriately using dH_2_O.

### 4.6. Biopharmacological Analyses

#### 4.6.1. Ethical Consideration

Blood was obtained from a healthy, non-medicated human donor after obtaining informed consent according to the declaration of Helsinki and after ethical review board (ERB) approval No. 2022/19269/MedAll/TRY, dated 12 August 2022.

#### 4.6.2. In Vitro Hemocompatibility

The hemocompatibility of PF/HA-QtN#AgNPs was assessed after their exposure to blood [23]. First, 3 mg equivalents of PF/HA-QtN#AgNPs were placed in culture plates made of polystyrene and stirred with 1x PBS (pH 7.4). A total of 4 mL of blood was then exposed to the materials for 30 min and agitated at 80 ± 3 rpm with a shaker set at 37 ± 1 °C. A Sysmex-K4500 automatic hematology analyzer (GMI Inc., Ramsey, MN, USA) was used to measure the total amount of hemoglobin (Hb) in the original blood sample. By determining the absorbance of dilute plasma using a diode array spectrophotometer (Elico, Hyderabad, India), it was possible to determine the amount of free Hb that was freed into the plasma following contact with the components in the whole sample [24].

Hemolysis (%) = (Free Hb/Total Hb) × 100

The blood samples were centrifuged at 1500 rpm for 10 min to obtain platelet-rich plasma (PRP) for an examination of platelet aggregation following exposure. From the initial and final blood samples, the platelet aggregation response to the agonists, i.e., collagen and adenosine diphosphate (ADP), was assessed in PRP. By means of an Asserachrom HPIA ELISA kit (Stago, Asnieres-sur-Seine, France), the platelet factor (PF4) was examined using a DIGISCAN Reader (ASYS HITECH Inc., Eugendorf, Austria) to measure the absorbance and a Microwin 2000 to process the data. For each experiment, blank polystyrene culture dishes that had been exposed to blood were utilized as a comparison.

#### 4.6.3. Cellular Uptake Studies

Caco-2 and HeLa cells were grown in DMEM supplemented with 10% FBS and 1% penicillin/streptomycin at 37 °C with 5% CO_2_ and 95% humidity. To eliminate nonspecific binds, cells were washed with 1x PBS (pH 7.4) for four hrs after being incubated with FITC-labeled PF/HA-QtN#AgNPs. Extracellular fluorescence was quenched using 0.4% trypan blue for 5 min. A Zeiss LSM710 confocal laser scanning microscope (CSLM) (Carl Zeiss Microscopy Deutschland GmbH, Oberkochen, Germany) was used to examine the cellular uptake of the compound. Using flow cytometry/fluorescence-activated cell sorting (FACS), the percentage of cellular uptake was calculated. To reduce the extracellular fluorescence, the cells were treated with a 0.4% trypan blue solution for 2 min after the incubation period and rinsed three times with 1x PBS (pH 7.4). The cells were then treated for two min with trypsin-EDTA (to detach the cells from the dish) before being suspended in 1x PBS (pH 7.4). FACS was used to calculate the proportion of cells that had taken up AgNPs after being excited with a 570 nm argon laser and detected with band-pass filter [16]. This proportion was calculated based on the formula given below. A total of 1000 cells were examined in each experiment using a BD FACSAria III cell sorter (BD Biosciences, San Jose CA, USA) to measure the suspensions. BD FACSDivaTM Software (BD Biosciences, San Jose CA, USA) was used to acquire and process the data.

$$\%\;cell\;viability=\frac{Abs\;of\;sample}{Abs\;of\;control}\times 100$$

#### 4.6.4. In Vitro Cytotoxicity

The MTT assay consists of the metabolic conversion of 3-(4,5-dimethylthiazol-2yl)-2,5, diphenyltetrazolium bromide to a colored formazan by living cells [29,30]. MTT was used to measure the cytotoxicity of PF/HA-QtN#AgNPs and free QtN (control). HeLa cells and Caco-2 cells were separately seeded in a 96-well tissue culture plate at a density of 5 × 10^3^ cells/well in 100 µL of DMEM with 10% FBS to test the viability of the cells. After 24 h at 37 °C with 5% CO_2_ and 95% humidity, cells that had reached 70–80% confluence were treated with different concentrations of PF/HA-QtN#AgNPs or free QtN (10, 20, 30, 40, 50, and 60 µg). Following the removal of the medium’s derivatives after 24 h, 200 µL of medium was added to 50 µL of MTT (2 mg·mL^−1^ in DMEM), and the mixture was incubated for 4 h under standard growth conditions. After removing the MTT after 4 h, 150 µL of DMSO was applied to each well. Using an automatic microplate absorbance reader (iMark, Bio-Rad Laboratories, Hercules, CA, USA), the resulting solution absorbance/optical density (OD) was instantly measured at 570 nm. OD values were calculated after blank subtraction (i.e., medium alone).

### 4.7. Statistics

The outcomes of each assay represent the averages of three independent replicates and are displayed as means ± standard deviations (SDs). The statistics were analyzed using a one-way analysis of variance (ANOVA); *p* < 0.05 was considered statistically significant.

## 5. Conclusions

In this study, PF/HA-QtN#AgNPs were prepared using a one-shot technique, and they improved the water solubility and stability of QtN. The drug (QtN) conjugate functioned as a stabilizing and reducing moiety. This method is easier since it skips additional chemical processes to attach the drug to the prepared NPs. To enhance the ability of AgNPs to target cancer cells, PF was further added to the stable QtN-AgNPs. Blood and NPs interact, causing cellular and humoral responses that might activate coagulation and/or fibrinolysis. As a result, the formation of biomaterials with enhanced hemocompatibility is required to improve tolerability and reduce adverse consequences. Compared to QtN in its free/pure form, PF/HA-QtN#AgNPs showed better dose-dependent toxicity toward well-established cancer cell lines. The prepared PF/HA-QtN#AgNPs also showed improved targeting efficacy through endocytosis mediated by the folate receptor and the HA receptor. This study implies that the delivery mechanism might greatly improve the intracellular accumulation of AgNPs and facilitate the efficient transport of QtN to the perinuclear or nuclear regions of cells. In the future, it would be interesting to test the cytotoxicity in standard healthy non-cancer human cell lines to check the specificity of the NPs before in vivo studies.

## Figures and Tables

**Table 1:** AgNPs in aqueous solution: zeta potential (ZP) and hydrodynamic sizes (PS) as functions of surface modifications.

Formulation Code	Complex	ZP (mV)	PS (Mean Diameter in nm)
1	HA-QtN@AgNPs	−42.33 ± 1.67	72.11± 1.31
2	PF/HA-QtN@AgNPs	−28.9 ± 1.98	131.22 ± 3.51

**Table 2:** List of identified FTIR bands, absorption wavenumbers, and assignments.

Formulation	Absorption (cm^−1^)	Chemical Groups	Compound Class
HA-QtN#AgNPs	3008.41	weak O-H stretching	alcohol
	2926. 48	C-H stretching	alkane
	2855.10	strong C-H stretching	amine salt
	1460.81	C-H bending	alkane
PEG-FA	3439.42	N-H stretching	primary amine
	2922.59	C-H stretching	alkane
	2094.32, 1735.65	C=O stretching	aldehyde
	1642.09	C=N stretching	imine/oxime
	1351.22	strong S=O stretching	sulfonamide
	1298.22, 1105.01	strong C-O stretching	ester
	948.81, 842.74	C=C bending	ester
PF/HA-QtN#AgNPs	3446.17	O-H stretching	alcohol
	1637.27	C=C stretching	alkene
	1462.74	C-H bending	alkene
	1202.07	Strong C-O stretching	alkyl aryl ether

## Data Availability

Data sharing is not applicable.

## Sample Availability

Samples of the compounds are not available from the authors.

## References

[1] Senapati S., Mahanta A.K., Kumar S., Maiti P. (2018). Controlled drug delivery vehicles for cancer treatment and their performance. Signal Transduct. Target. Ther..

[2] Moideen M.M.J., Alqahtani A., Venkatesan K., Fazil Ahmad F., Krisharaju K., Gayasuddin M., Shaik R.A., Ibraheem K.M.M., Salama M.E.M., Abed S.Y. (2020). Application of the Box-Behnken design for the production of soluble curcumin: Skimmed milk powder inclusion complex for improving the treatment of colorectal cancer. Food Sci. Nutr..

[3] Gomes H.I.O., Martins C.S.M., Prior J.A.V. (2021). Silver Nanoparticles as Carriers of Anticancer Drugs for Efficient Target Treatment of Cancer Cells. Nanomaterials.

[4] Ashraf S., Abbasi A.Z., Pfeiffer C., Hussain S.Z., Khalid Z.M., Gil P.R., Parak W.J., Hussain I. (2013). Protein-mediated synthesis, pH-induced reversible agglomeration, toxicity and cellular interaction of silver nanoparticles. Colloids Surf. B.

[5] Arvaniti O.S., Samaras Y., Gatidou G., Thomaidis N.S., Stasinakis A.S. (2019). Review on fresh and dried figs: Chemical analysis and occurrence of phytochemical compounds, antioxidant capacity and health effects. Food Res. Int..

[6] Panche A.N., Diwan A.D., Chandra S.R. (2016). Flavonoids: An overview. J. Nutr. Sci..

[7] Khan F., Niaz K., Maqbool F., Ismail Hassan F., Abdollahi M., Nagulapalli Venkata K.C., Nabavi S.M., Bishayee A. (2016). Molecular Targets Underlying the Anticancer Effects of Quercetin: An Update. Nutrients.

[8] Rivas B.L., Urbano B.F., Sánchez J. (2018). Water-Soluble and Insoluble Polymers, Nanoparticles, Nanocomposites and Hybrids with Ability to Remove Hazardous Inorganic Pollutants in Water. Front. Chem..

[9] Kobayashi T., Chanmee T., Itano N. (2020). Hyaluronan: Metabolism and Function. Biomolecules.

[10] Serri C., Quagliariello V., Iaffaioli R.V., Fusco S., Botti G., Mayol L., Biondi M. (2019). Combination therapy for the treatment of pancreatic cancer through hyaluronic acid-decorated nanoparticles loaded with quercetin and gemcitabine: A preliminary in vitro study. J. Cell Physiol..

[11] Mansoori-Kermani A., Khalighi S., Akbarzadeh I., Niavol F.R., Motasadizadeh H., Mahdieh A., Jahed V., Abdinezhad M., Rahbariasr N., Hosseini M. (2022). Engineered hyaluronic acid-decorated niosomal nanoparticles for controlled and targeted delivery of epirubicin to treat breast cancer. Mater. Today Bio.

[12] Pang X., Lu Z., Du H., Yang X., Zhai G. (2014). Hyaluronic acid-quercetin conjugate micelles: Synthesis, characterization, in vitro and in vivo evaluation. Colloids Surf. B.

[13] Juhaščik M., Kováčik A., Huerta-Ángeles G. (2022). Recent Advances of Hyaluronan for Skin Delivery: From Structure to Fabrication Strategies and Applications. Polymers.

[14] Mohamed J.M., Kavitha K., Ruckmani K., Shanmuganathan S. (2020). Skimmed milk powder and pectin decorated solid lipid nanoparticle containing (SLN) soluble curcumin used for the treatment of colorectal cancer. J. Food Process Eng..

[15] Martín-Sabroso C., Torres-Suárez A.I., Alonso-González M., Fernández-Carballido A., Fraguas-Sánchez A.I. (2021). Active Targeted Nanoformulations via Folate Receptors: State of the Art and Future Perspectives. Pharmaceutics.

[16] Bharadwaj K.K., Rabha B., Pati S., Choudhury B.K., Sarkar T., Gogoi S.K., Kakati N., Baishya D., Kari Z.A., Edinur H.A. (2021). Green Synthesis of Silver Nanoparticles Using Diospyros malabarica Fruit Extract and Assessments of Their Antimicrobial, Anticancer and Catalytic Reduction of 4-Nitrophenol (4-NP). Nanomaterials.

[17] Arif W., Rana N.F., Saleem I., Tanweer T., Khan M.J., Alshareef S.A., Sheikh H.M., Alaryani F.S., Al-Kattan M.O., Alatawi H.A. (2022). Antibacterial Activity of Dental Composite with Ciprofloxacin Loaded Silver Nanoparticles. Molecules.

[18] Thapa P., Li M., Karki R.M., Bio M., Rajaputra P., Nkepang G., Woo S., You Y. (2017). Folate-PEG Conjugates of a Far-Red Light-Activatable Paclitaxel Prodrug to Improve Selectivity toward Folate Receptor-Positive Cancer Cells. ACS Omega.

[19] Mohamed J.M., Alqahtani A., Ahmad F., Krishnaraju V., Kalpana K. (2021). Pectin co-functionalized dual layered solid lipid nanoparticle made by soluble curcumin for the targeted potential treatment of colorectal cancer. Carbohydr. Polym..

[20] Zafar N., Uzair B., Niazi M.B.K., Menaa F., Samin G., Khan B.A., Iqbal H., Menaa B. (2021). Green Synthesis of Ciprofloxacin-Loaded Cerium Oxide/Chitosan Nanocarrier and its Activity Against MRSA-Induced Mastitis. J. Pharm. Sci..

[21] Perugini V., Schmid R., Mørch Ý., Texier I., Brodde M., Santin M. (2022). A multistep in vitro hemocompatibility testing protocol recapitulating the foreign body reaction to nanocarriers. Drug Deliv. Transl. Res..

[22] Zou Y., Xiao F., Song L., Sun B. (2021). A folate-targeted PEGylated cyclodextrin-based nanoformulation achieves co-delivery of docetaxel and siRNA for colorectal cancer. Int. J. Pharm..

[23] Akhtar N., Akhtar N., Menaa F., Alharbi W., Alaryani F.S.S., Alqahtani A.M., Ahmad F. (2022). Fabrication of Ethosomes Containing Tocopherol Acetate to Enhance Transdermal Permeation: In Vitro and Ex Vivo Characterizations. Gels.

[24] Mohamed J.M.M., Mahajan N., El-Sherbiny M., Khan S., Al-Serwi R.H., Attia M.A., Altriny Q.A., Arbab A.H. (2022). Ameliorated Stomach Specific Floating Microspheres for Emerging Health Pathologies Using Polymeric Konjac Glucomannan-Based Domperidone. BioMed Res. Int..

[25] Laloy J., Minet V., Alpan L., Mullier F. (2014). Impact of Silver Nanoparticles on Haemolysis, Platelet Function and Coagulation. Nanobiomedicine.

[26] Fernández M., Javaid F., Chudasama V. (2017). Advances in targeting the folate receptor in the treatment/imaging of cancers. Chem. Sci..

[27] Narvekar M., Xue H.Y., Eoh J.Y., Wong H.L. (2014). Nanocarrier for poorly water-soluble anticancer drugs—Barriers of translation and solutions. AAPS PharmSciTech.

[28] Moideen Muthu Mohamed J., Khan B.A., Rajendran V., El-Sherbiny M., Othman G., Bashir Ahmed Hussamuldin A., Hamed Al-Serwi R. (2022). Polymeric ethosomal gel loaded with nimodipine: Optimisation, pharmacokinetic and histopathological analysis. Saudi Pharm. J..

[29] Srividya G., Jainaf Nachiya R.A.M., Ebrahim D., El-Sagheer A.M., Kayarohanam S., Janakiraman A.K., Khan J., Mohamed J.M.M. (2022). Comparative study of semi-solid bases of naproxen: Pharmaceutical technology aspects. Int. J. Appl. Pharm..

[30] Manju S., Sreenivasan K. (2012). Gold nanoparticles generated and stabilized by water soluble curcumin–polymer conjugate: Blood compatibility evaluation and targeted drug delivery onto cancer cells. J. Colloid Interface Sci..

